# Preventing corneal blindness: working with communities

**Published:** 2009-12

**Authors:** Hannah Faal

**Affiliations:** Programme Development Adviser: Health Systems, Sightsavers International, 21 Nii Nortei Ababio Road, PO Box KIA 18190, Airport, Accra, Ghana.

**Figure:**
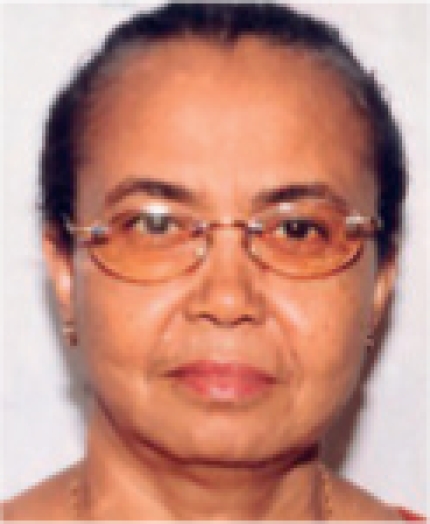


Preventing corneal blindness in the community involves action by the community itself, as well as actions by government and non-governmental organisations in the form of health and development services. In order to be effective, eye health workers need to understand how all of the above can work together.

**Figure:**
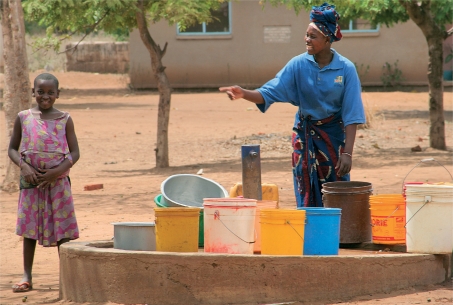
Community development projects help to reduce the most common causes of corneal blindness. TANZANIA.

Lance Bellers/Sightsavers International

Prevention of corneal blindness takes place at three levels:

1. **Primary prevention:** Actions or interventions taken to prevent the onset of disease
2. **Secondary prevention:** Actions taken to prevent complications and/or the development of visual disability due to an existing disease
3. **Tertiary prevention:** After the immediate problem has been addressed by surgery or other treatment, actions to restore function or reduce existing disability from disease complications, i.e. corneal transplantation (see page 44).

Right up to the point when someone is seen by an eye care worker or admitted to hospital, the community will influence what happens.

Consider ophthalmia neonatorum as an example. As an eye care worker, you may have little control over the following:

- **the risk factors and immediate medical causes**, e.g. parents' sexual behaviour and the presence of *Neisseria gonorrhoea*
- **the contributory and social factors**, e.g. the lack of antibiotic drops in the labour ward, poverty associated with dangerous work, poor access to water and sanitation, or a community's preference for traditional medicines.

However, the community has the potential to influence most of these factors, either through change in the behaviour of individuals, or by lobbying for improvements at the community level.

**Figure:**
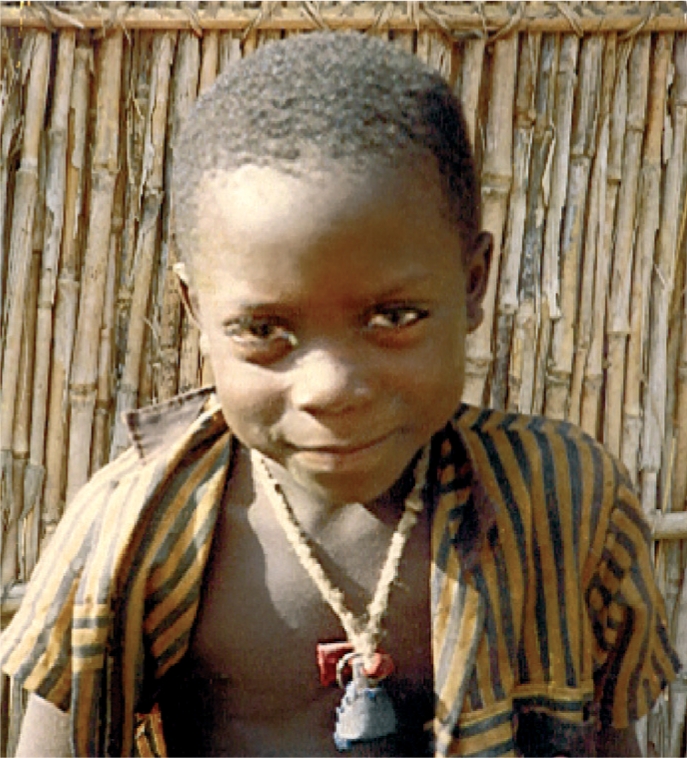
Traditional remedies, such as the one around this boy's neck, contribute to delays in seeking medical treatment. THE GAMBIA

Sightsavers International

The eye care worker's role, in particular when designing or participating in programmes to reduce corneal blindness, should be to inform and assist the community to address whichever of the above factors are relevant. This will allow the community to become an active partner in the prevention of corneal blindness.

## First steps

A successful corneal blindness prevention programme **does not:**

- focus solely on individual diseases
- ignore the perceptions, knowledge, and abilities of the community
- work in isolation from other services in the health system.

A successful corneal blindness prevention programme **does:**

- address the overall causes of corneal blindness in the community
- aim to understand the community, build on their existing knowledge, and encourage and support them to campaign for better services
- understand the health and development services available in the community with a view to supporting them and making the best possible use of them.

Whether you are planning to improve a prevention programme or designing a new one, it is helpful to learn as much as possible about what the community needs and how they may be able to support your programme.

Doing so ensures that as many people as possible are involved right from the start.

It is a good idea to do a situation analysis, which will help to clarify what you know about the community and identify any gaps in your knowledge (which you will then need to fill).

Here are some questions to get you started:

- What are the community's knowledge and perceptions regarding the causes and treatment of corneal blindness?
- What are the existing and traditional methods of communication within the community? How can these be used to transmit new health messages?
- How will the community's knowledge and perceptions influence the content of health messages and how they are presented?
- What skills exist within the community that may be used to support the programme?
- What are the first points of contact for care: homes, schools, traditional healers, or pharmacists/chemist shops? What first aid is usually practiced?

## Primary prevention

Primary prevention of corneal blindness is particularly relevant for the following causes:

- vitamin A deficiency and measles
- ophthalmia neonatorum
- trachoma
- eye injuries.

There are many social factors associated with corneal disease, such as poverty, inadequate water supply and sanitation, poor nutrition, and dangerous agricultural practices. Other contributing factors may include the high cost or unavailability of medicines or safety goggles. A good programme should support the community to obtain the health care and other services it needs, either by mobilising the community's own resources or by lobbying government for help.

To address the immediate medical causes and risk factors, the programme should provide health education about risk factors and how to avoid them, as well as information about what to do and where to go for help if an eye problem develops.

Support for these activities may be possible by closely collaborating with the health promotion unit of the local or national health system.

Good communication is essential. Use what you have learnt from the situation analysis to plan communication activities, for example by using existing and community-friendly methods. In urban areas, use the media and billboards. In rural areas, a meeting of the village elders may be more effective. Integration of eye health messages into the school curriculum is another possibility.

## Secondary prevention

The cornea is transparent and sensitive to pain. As a result, patients with corneal disease or injuries are usually in pain and may suffer from photophobia; their eyes may water and they may have blurred vision. These all prompt the patient or carer (in the case of a child) to take action early.

Because of the pain, people may self-medicate, either with harmful medicines obtained from family members or from nearby care providers such as traditional healers or local pharmacists/chemist shops.

These early attempts at seeking care may be harmful, but also delay the process of obtaining correct treatment from the nearest medical facility. Both factors - wrong management and delay - may contribute more to corneal opacity and visual loss than the original cause.

**Figure:**
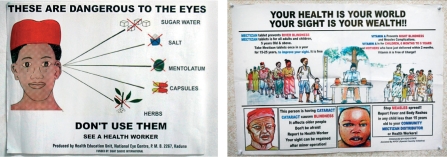
Eye health posters used to inform the community. THE GAMBIA

Sightsavers International (2)

## Fighting corneal blindness by strengthening health systems

Understanding the health systems which already serve the community will help to ensure that new programmes make the best use of what is available and don't overload existing services. With careful thought, it may even be possible for a programme to contribute to the existing health systems, leaving them stronger and better able to serve the community in future.

Many health and community development programmes already in existence, such as measles immunisation, perinatal care, nutrition, water supply, and sanitation, make a significant contribution to reducing the most common causes of corneal blindness. It is important to support these programmes by informing policy makers and funding agencies of their impact on the prevention of blindness, as this will increase the motivation of those involved and may improve the prospects for continued political and financial support.

The following aspects of existing health systems can be strengthened with your help.

### The health work force

- Work with the community to identify individuals who can provide home-based care and training. These could include retired professional people such as health workers or teachers who have returned to live in their communities. Support them either directly or through existing primary health care structures.
- Collaborate with existing health workers, traditional or not. For example, ensure that they are aware of the dangers of steroid eye drops and that they understand why it is important to instil antibiotic or antiseptic drops in newborn babies' eyes.
- Teach all health workers to diagnose and refer corneal pathology early.

### Medical products and vaccines

- Ensure that basic items, such as torches and antibiotic/antiseptic drops, are available at points of need, e.g. in labour wards, with traditional birth attendants, or in schools.
- Support existing efforts to provide vaccines and ensure cold chains.

### Health information

- Gather information about the impact of corneal disease and trauma in the community. For example, ensure that ophthalmia neonatorum is a notifiable disease and record the number of children with measles or xerophthalmia.
- Gather evidence about the effectiveness of community-focused intervention measures, such as water and sanitation programmes, immunisation campaigns, or free health care for children.
- Use your evidence to improve programme design and service delivery, and to lobby the authorities to maintain and strengthen these programmes.

### Health financing

- Work with the community to ensure that emergency eye care for corneal infections or trauma, particularly in children, is free and that cost does not restrict access to treatment.

### Leadership and governance

- Work with the decision-making bodies responsible for the local community's development and health. Encourage them to allocate resources to measures such as latrine construction and home-based care.
- Encourage communities to take the lead on health matters, for example by working with community development groups. Ordinary people can take responsibility for a range of interventions, from household-led health activities such as face washing to demanding better services.
- Support and encourage communication between the community and decision makers within the health system, as well as between different groups or specialties in the health system.

## Conclusion

As an eye health worker designing or implementing a programme to prevent corneal blindness in the community, you should understand both the medical causes of corneal scarring, and the non-medical and social factors that lead to corneal blindness. You should recognise the potential of the community to be involved and actively seek out ways to ensure their involvement.

It is vital to understand the impact of development programmes led by other government departments (education, agriculture, water resources, community development, and justice) on the prevention of corneal blindness. Eye workers must support and work with these initiatives.

In order to do this well, you, as an eye health worker, should develop non-medical skills such as communication, negotiation, advocacy, and the ability to foster community engagement.

